# Supplementary material for: Decellularization of Full Heart—Optimizing the Classical Sodium-Dodecyl-Sulfate-Based Decellularization Protocol
Source: Bioengineering (Basel). 2022 Apr 1;9(4):147. doi: 10.3390/bioengineering9040147 (PMC9032179; doi:10.3390/bioengineering9040147)
Supplement: Supplementary file 1 [file bioengineering-09-00147-s001.zip › Supplementary Figures.pdf]

bioengineering

Article

Decellularization of Full Heart—Optimizing the Classical Sodium-Dodecyl-Sulfate-Based Decellularization Protocol

Reem Al-Hejailan [1], Tobias Weigel [2], Sebastian Schürlein [2], Constantin Berger [2], Futwan Al-Mohanna [1] and Jan Hansmann [2,*]

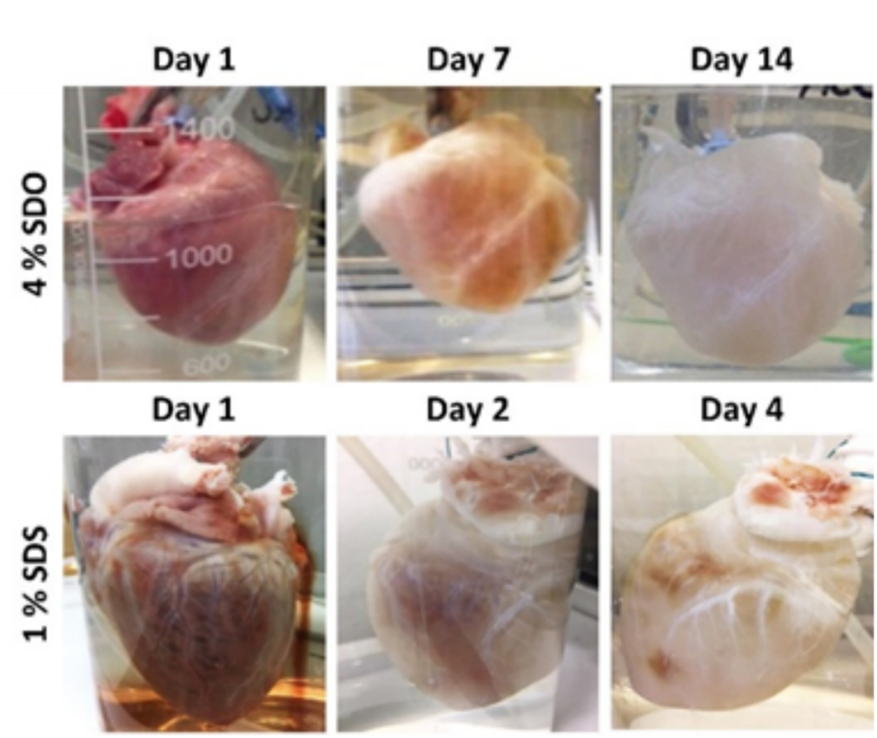

Supplementary Figure S1 Decellularization of the whole heart. Images of the decellularization processes of the whole heart using protocol 1 (4 % SDO) or protocol 2 (1 % SDS). Different time points for each protocol are shown (protocol 1: day 1, 7, 14; protocol 2: 1, 2, 4) to demonstrate the speed of decellularization. Using protocol 2, decellularization was faster and decellularized parts could already be detected at day 4, while protocol 2 did not lead to a visible decellularization before day 14.

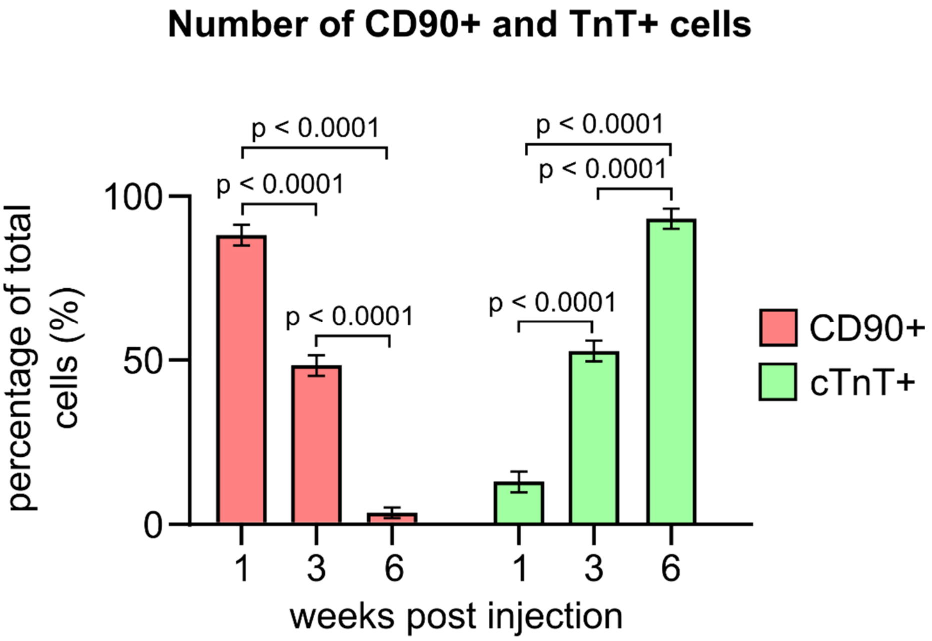

Supplementary Figure S2 Quantification of cell types of the repopulated cardiac patch. Percentage of total cells expressing the hMSC marker CD90 or the cardiac marker cTnT in the re-seeded heart patch 1, 3 and 6 weeks post seeding. Data represent mean ± SEM (n = 3 analysed sections for each time point); **** $p < 0.0001$.
